# Case Report: Congenital gingival angiomatosis in a newborn lamb

**DOI:** 10.3389/fvets.2025.1702299

**Published:** 2025-11-18

**Authors:** D. Zapico, V. Pérez, M. Criado, P. Mendívil, M. Silva, J. Benavides, M. C. Ferreras, J. Espinosa

**Affiliations:** 1 Departamento de Sanidad Animal, Facultad de Veterinaria, Universidad de León, León, Spain; 2 Instituto de Ganadería de Montaña, CSIC-ULE, León, Spain

**Keywords:** lamb, gingiva, vascular malformation, hamartoma, angiomatosis

## Abstract

Proliferative vascular disorders are rare congenital tumor-like lesions that arise from anomalies in blood vessel development. A newborn Assaf lamb presented with a diffuse, multinodular, dark-red overgrowth of the periodontal gingiva, accompanied by refusal to suckle. The animal developed diarrhea and died 2 days after birth. Necropsy was performed, and histopathological examination of the oral lesions revealed a marked expansion of the gingival stroma by multiple, closely packed, small- to medium-sized vascular channels. These were lined by a single layer of von Willebrand factor- and vimentin-positive endothelial cells and were partially surrounded by alpha smooth muscle actin (*α*-SMA) - and vimentin-positive spindle cells, consistent with well-differentiated capillaries and post-capillary venules. Taken together, these findings were consistent with diffuse vascular gingival hamartomatosis/angiomatosis. The congenital nature and distribution of the lesions are supportive of a malformation of the periodontal vasculature.

## 1 Introduction

Proliferative gingival lesions in lambs and adult sheep are most commonly associated with orf virus infection (contagious ecthyma) (1, 2). Other causes of gingival swelling include periodontitis (3), trauma, dentigerous cysts (4, 5), and, less frequently, neoplasia. The most common oral tumors in sheep, although rare by themselves, are squamous cell carcinoma and, to a lesser extent, fibrosarcoma (6–9), whereas other neoplasms such as ossifying fibroma (10), odontoameloblastoma (11), or hemangioma (12) have been sporadically reported.

Non-neoplastic proliferative vascular lesions (e.g., vascular hamartomas, arteriovenous malformations, and angiomatosis) are infrequent in domestic animals (13, 14). Microscopic similarity and overlap in terminology often make the histopathological classification of these disorders challenging. In practice, the term “vascular hamartoma” has been most widely used (15). Gingival vascular hamartomas have been reported mainly in calves and, less frequently, in cats. However, their presentation is usually focal, small in size, without extension to the teeth, and without interference with suckling or feeding (16–24). A case of vascular hamartoma has also been described in the skin of a Polwarth lamb (25). In the present study, we report a neonatal Assaf lamb with diffuse vascular proliferation extensively involving the gingival region. To the authors’ knowledge, this is the first report of a vascular malformation of this severity and distribution in the gingiva of a lamb.

## 2 Case description

A male newborn Assaf lamb from an intensive dairy farm of this breed showed apathy, anorexia, and refusal to suckle immediately after birth. Within the first hours of life, the animal developed progressive weakness and diarrhea and ultimately died at 2 days of age. The carcass was submitted to the Diagnostic Pathology Service of the University of León for *post-mortem* examination.

## 3 Diagnostic assessment

**Figure 1:**
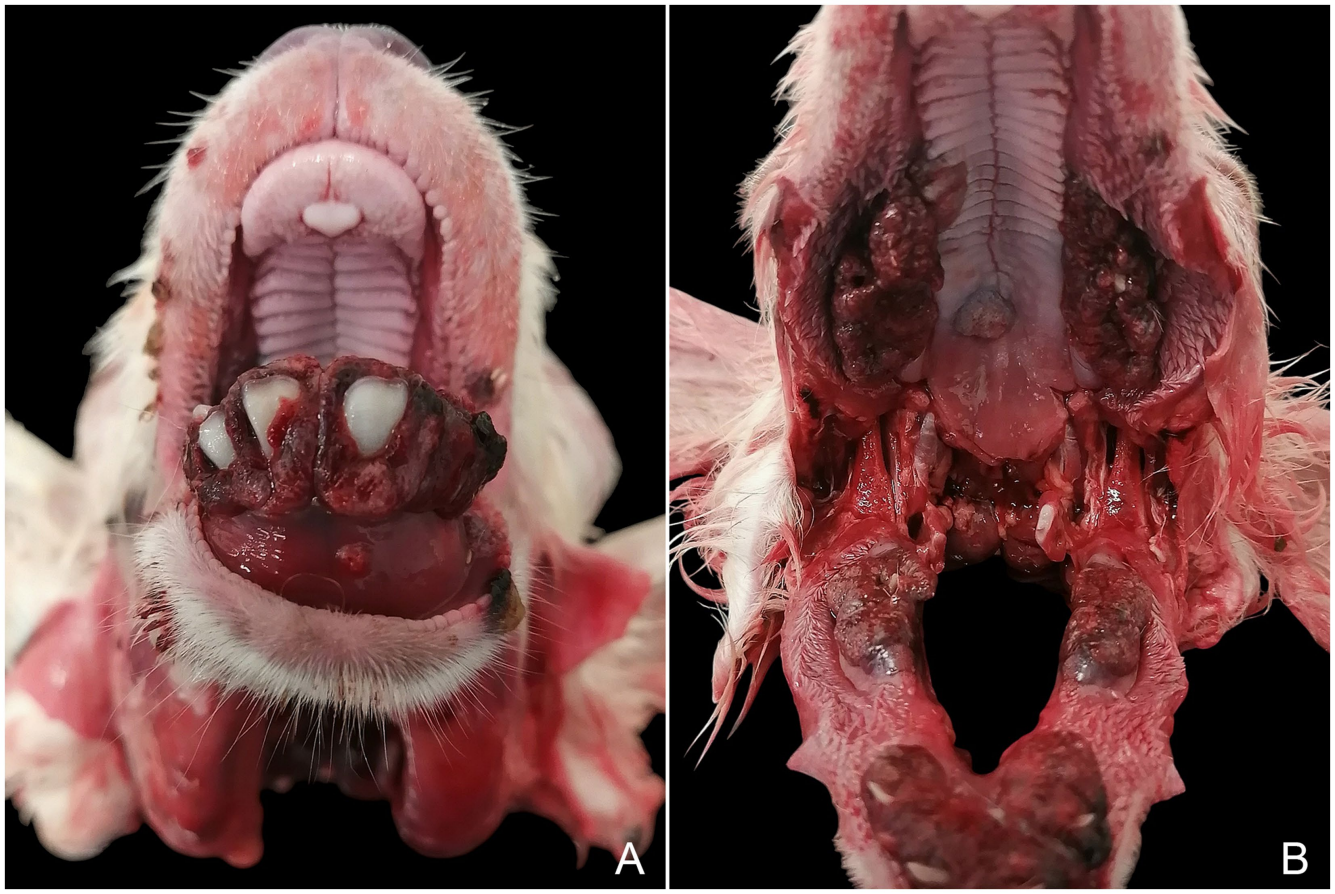
Maxilla and mandible. Exophytic, multinodular enlargement with dark-red discoloration of the gingiva, partially to completely covering the teeth **(A,B)**, while sparing the dental pad **(A)** and the diastema **(B)**.

At necropsy, the animal presented with severe, diffuse, exophytic overgrowth, and dark-red discoloration of the periodontal gingiva, which partially or completely covered the incisors, premolars, and molars in both the maxilla and the mandible (Figures 1A,B). These changes were absent from the dental pad (Figure 1A) and the diastema (Figure 1B). Another lesion observed during *post-mortem* examination was segmental distension of the small intestine, containing mild to moderate amounts of gas and liquid content, consistent with catarrhal enteritis. This condition was most likely secondary to insufficient colostrum intake caused by impaired or painful suckling. Therefore, it can be concluded that catarrhal enteritis represented the final cause of death in this lamb.

Tissue samples of the entire mandible and maxilla (including teeth, gingiva, and alveolar bone), as well as from the other organs of the animal, were fixed in 10% neutral phosphate-buffered formalin. Subsequently, the mandibular and maxillary samples showing proliferative lesions were treated with a demineralization solution composed of sodium citrate and formic acid (50/50%, v/v) for 7 days. Afterward, samples were carved, dehydrated through a graded alcohol series and xylene treatment, and embedded in paraffin wax. Sections 3-μm thick were obtained and stained with Harris’s hematoxylin and eosin for histopathological examination.

Immunohistochemical analysis of von Willebrand factor (A008229-2, Dako-Agilent Technologies, Santa Clara, CA, USA, 1:200), alpha smooth muscle actin (α-SMA) (M0851, Dako-Agilent Technologies, Santa Clara, CA, USA, 1:100 dilution), and vimentin (M0725, Dako-Agilent Technologies, Santa Clara, CA, USA, 1:1,000 dilution) was further performed. The immunohistochemical procedure was carried out as described elsewhere (26). Briefly, heat-mediated antigen retrieval was performed with PT Link® system (Dako-Agilent Technologies, Santa Clara, CA, USA), using pH 6.0 target retrieval solution for vimentin and pH 9.0 solution for *α*-SMA, for 20 min at 95 °C. Sections were then submerged for 30 min in 3% H_2_O_2_ in methanol solution at room temperature to block endogenous tissue peroxidase. For the von Willebrand factor, antigen retrieval was performed for 15 min at room temperature using proteinase K (Dako-Agilent Technologies, Santa Clara, CA, USA) after deparaffination, hydration, and peroxidase blockage. Tissue slides were then incubated overnight with the primary antibody in a humidified chamber at 4 °C. Afterward, sections were exposed to appropriate anti-mouse or anti-rabbit secondary antibody for 40 min at room temperature using the EnVision System® (Dako-Agilent Technologies, Santa Clara, CA, USA). Immunostaining was developed using 3,3-diaminobenzidine (Dako-Agilent Technologies, Santa Clara, CA, USA) as a chromogenic substrate, and slides were counterstained with hematoxylin. Appropriate species- and isotype-matched immunoglobulins were used as a control. These included sections with an isotype control for the primary antibody and the omission of the primary antibody. As positive controls, sections from the jejunum of clinically healthy sheep were used.

**Figure 2:**
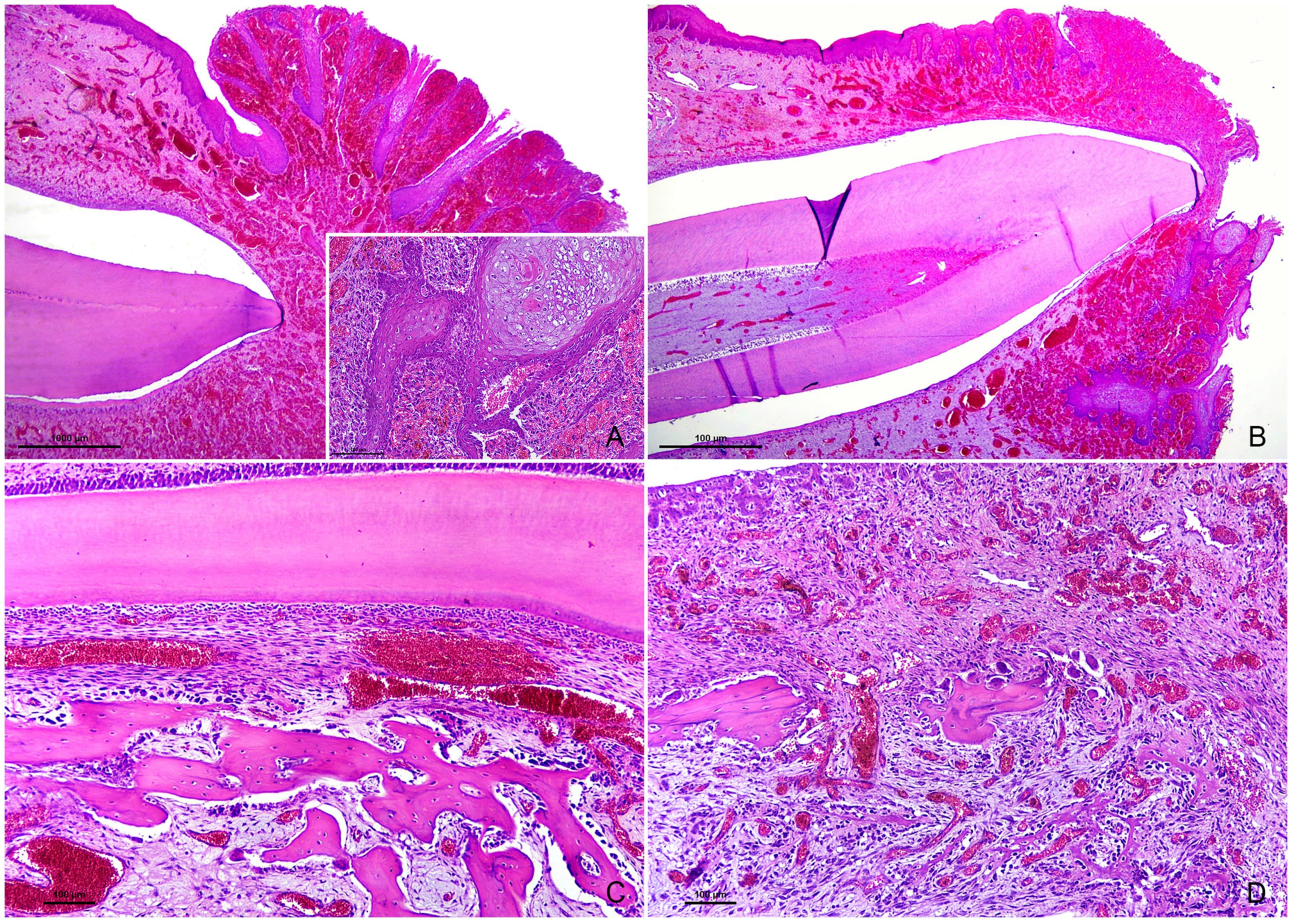
Tissue sections of the teeth, periodontium, gingiva, and alveolar bone. Hematoxylin and eosin. **(A)** Exophytic expansion of the free gingiva due to extensive vascular proliferation within the stroma, completely covering the tooth. The epithelium is markedly ulcerated and hyperplastic, with prominent rete ridges. Magnification: 40×. Inset: epithelial swelling and hydropic (ballooning) degeneration (top right). Magnification: 200×. **(B)** Decidual tooth with normal dental pulp, dentin, and an artefactual space resulting from enamel loss during tissue processing. The surrounding gingiva shows changes similar to those described in **(A)**. Magnification: 40×. **(C)** Periodontal ligament fibers and fibroblasts are oriented parallel to the long axis of the tooth. A focal hemorrhage is also present. Magnification: 200×. **(D)** Woven bone fragments with adjacent osteoclasts within Howship’s lacunae (center), consistent with osteolysis and alveolar bone resorption, accompanied by moderate numbers of osteoblasts lining thin, irregular, newly formed bony trabeculae (bottom). Magnification: 200×.

Histopathological examination revealed a marked exophytic expansion of the free gingiva resulting from the proliferation of numerous disorganized, densely packed, small- to medium-sized vascular channels filled with blood in the stroma, partially or completely covering the teeth. Proliferating vascular channels were embedded in mild to moderate fibrous stroma and had irregular lumina lined by a single layer of flattened endothelial cells. These vascular structures extended from the alveolar crest toward the base of the overlying epithelium. The epithelium was markedly ulcerated and hyperplastic, with prominent anastomosing rete ridges (Figures 2A,B). Multifocal areas of epithelial swelling and hydropic change (ballooning degeneration) were also observed (Figure 2A, inset). Additional findings included an atypical orientation of periodontal ligament fibers and mesenchymal cells parallel to the long axis of the tooth (Figure 2C), as well as extensive areas of alveolar bone remodeling, characterized by increased osteoclastic activity and osteolysis (bone resorption) concurrent with immature bone formation by variable numbers of osteoblasts (Figure 2D). These changes were interpreted as physiologic processes associated with normal tooth eruption rather than true pathologic lesions.

**Figure 3:**
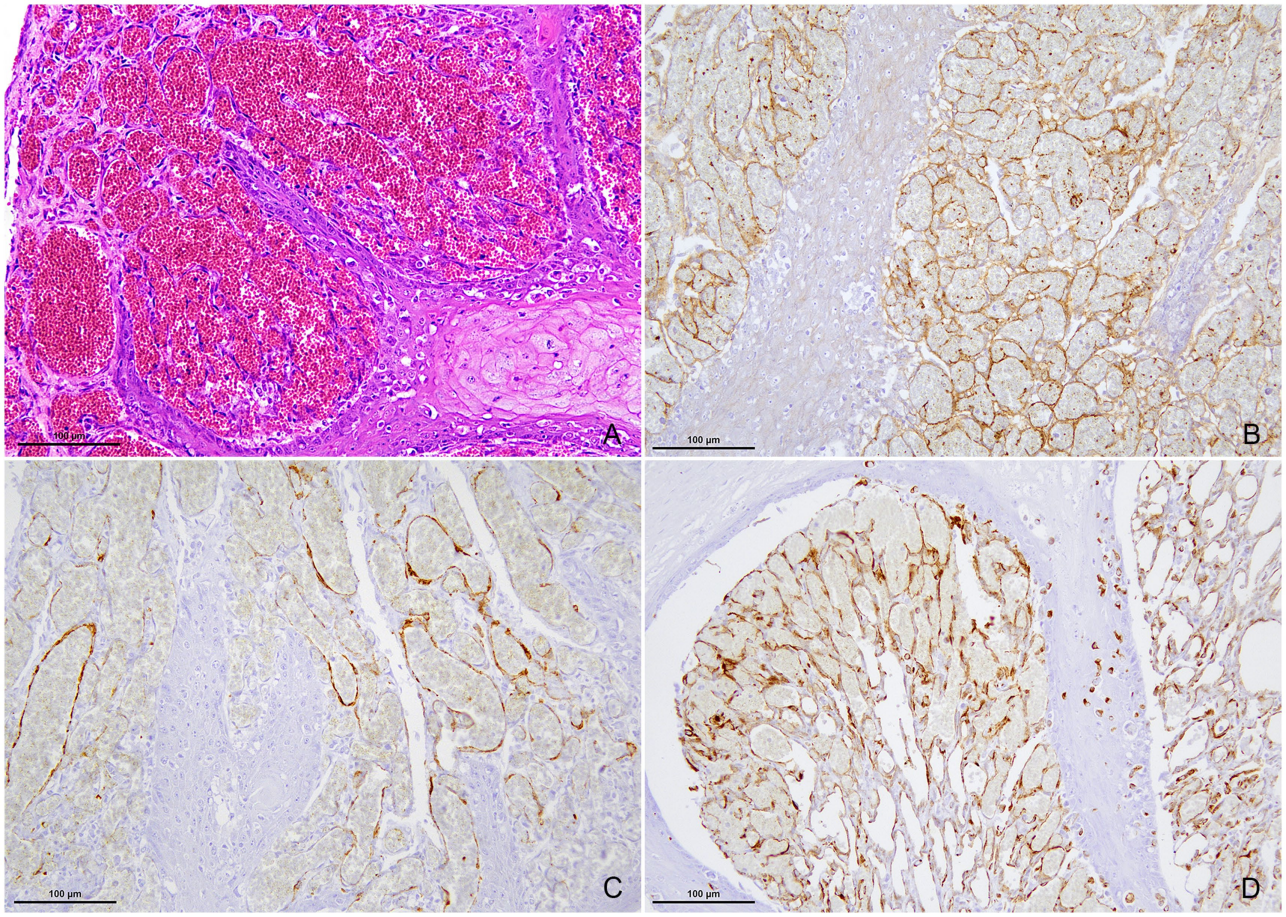
High magnification microphotographs of the proliferating vascular structures. **(A)** Hematoxylin and eosin. Closely packed vascular channels with irregular lumen, lined by a single layer of flat endothelial cells. **(B)** Immunohistochemistry (IHC) for the von Willebrand factor. Endothelial cells show strong immunoreactivity for von Willebrand factor. **(C)** IHC for α-SMA. Vascular endothelium displays variable α-SMA labeling, while larger vessels are partially surrounded by α-SMA-positive spindle-shaped cells. **(D)** IHC for vimentin. Both perivascular walls and endothelium are positive for vimentin. Magnification: 200×.

Immunohistochemical analysis revealed that the endothelium of the proliferative vascular channels (Figure 3A) was strongly positive for von Willebrand factor (Figure 3B) and vimentin in the cytoplasm, with variable immunoreactivity for *α*-SMA (Figure 3C). Larger-caliber vascular structures were partially surrounded by elongated spindle-shaped cells exhibiting cytoplasmic α-SMA (Figure 3C) and vimentin (Figure 3D) immunolabeling, consistent with perivascular smooth muscle cells and pericytes. Overall, these vascular channels were considered compatible with structurally normal capillaries and post-capillary venules.

## 4 Discussion

Congenital hemangiomas have been described in several animal species, including sheep (13, 14, 27). These benign vascular tumors are composed of variably sized, blood-filled vascular spaces lined by a single layer of flat endothelial cells, but they lack the smooth muscle and pericytic support characteristic of normal vessels (13, 14). In the present case, the vascular channels were partially supported by *α*-SMA- and vimentin-positive perivascular cell elements, compatible with smooth muscle cells or pericytes, suggesting a non-neoplastic process. However, some reports indicate that hemangiomas may occasionally display pericytic components, and therefore their absence cannot be considered a definitive diagnostic criterion (15). Furthermore, hemangiomas are typically solitary and well-circumscribed masses, which does not match the multifocal and periodontal distribution of the gingival lesions observed here. Malignant vascular tumors, such as hemangiosarcoma, may occur as multiple lesions, but they usually exhibit invasive growth and are composed of irregular vascular channels lined by one or more layers of spindle cells with varying degrees of atypia and mitotic activity (13, 14). These histological features were not present in the current case.

The term hamartoma refers to a non-neoplastic overgrowth of mature tissue elements native to the site of origin (13). Vascular hamartomas appear as single, well-demarcated, expansile masses composed of different proportions of capillaries, arterioles, and venules (13, 14, 28). They are congenital in nature and result from developmental anomalies in blood vessels, leading to an architectural disarray of the local vasculature (15). In this case, the disorganized proliferation of vascular channels lined by flat endothelial cells and variably supported by perivascular cell elements, compatible with mature capillaries and post-capillary venules, was consistent with the microscopic features of a vascular hamartoma. However, the widespread, multifocal distribution of the gingival lesions does not entirely conform to the classic definition of this condition. On the other hand, the concept of angiomatosis encompasses a heterogeneous group of non-neoplastic vascular proliferations, including specific syndromes such as bovine cutaneous angiomatosis and progressive angiomatosis of dogs and cats (13, 14, 29). More broadly, the term can be applied to describe multiple vascular hamartomas within the same tissue or anatomical region (30). Accordingly, the multifocal distribution of the gingival lesions in this lamb provides a rational basis for the diagnosis of gingival angiomatosis.

The exact etiology or pathogenesis of this vascular proliferation remains uncertain. Nevertheless, the topographic distribution of the lesions strongly suggests an origin related to periodontal structures. Although the vascular anatomy of the ovine periodontium has not been well characterized, studies in other species indicate that the free gingiva is supplied by supraperiosteal arterioles, periodontal ligament arterioles, and vessels emerging from the alveolar crest. These anastomose with descending venules beneath the sulcular epithelium form the dento-gingival or crevicular plexus (31–34). A congenital vascular malformation of this plexus could explain both the distribution of the gingival lesions and the absence of proliferations in edentulous areas such as the dental pad.

Finally, contagious ecthyma should also be considered among the differential diagnoses. Atypical proliferative lesions caused by orf virus have been reported in the gingiva of sheep and lambs. These are characterized by marked papillomatous epithelial hyperplasia supported by a highly vascularized stroma, changes that have been associated with a viral VEGF-like protein (35). Other common microscopic features include epithelial cell swelling, vacuolization, ballooning degeneration, and eosinophilic, intracytoplasmic inclusion bodies (36). While the farm where this case occurred had a history of contagious ecthyma, molecular confirmation of the virus was not possible. Nevertheless, the congenital nature of the vascular proliferations described here, together with their extensive distribution in a 48-h-old lamb, strongly argues against a viral etiology, as lesions of this magnitude could not have developed within such a short postnatal period. Additionally, no poxviral inclusion bodies were identified in the sections examined.

In conclusion, diffuse gingival angiomatosis in neonatal lambs appears to be an exceptionally rare condition, with no comparable reports currently available in the veterinary literature. The congenital nature and multifocal distribution of the lesions, together with their location restricted to periodontal structures, highlights a unique vascular malformation process rather than true neoplastic proliferation. This case expands the spectrum of vascular anomalies described in ruminants and underscores the need for further studies on the vascular anatomy and developmental disorders of the ovine periodontium.

## Data Availability

The original contributions presented in the study are included in the article/supplementary material, further inquiries can be directed to the corresponding author.
